# Evaluation of intrarenal hypoperfusion and renal congestion assessed by intrarenal Doppler ultrasonography in dogs with myxomatous mitral valve disease

**DOI:** 10.1093/jvimsj/aalag273

**Published:** 2026-11-13

**Authors:** Yuki Iwai, Tomoya Morita, Hiroki Terukina, Masahiro Yamasaki

**Affiliations:** Laboratory of Veterinary Small Animal Internal Medicine, Cooperative Department of Veterinary Medicine, Faculty of Veterinary Medicine, Iwate University, Morioka 020-8550, Japan; Laboratory of Veterinary Small Animal Internal Medicine, Cooperative Department of Veterinary Medicine, Faculty of Veterinary Medicine, Iwate University, Morioka 020-8550, Japan; Laboratory of Veterinary Small Animal Internal Medicine, Cooperative Department of Veterinary Medicine, Faculty of Veterinary Medicine, Iwate University, Morioka 020-8550, Japan; Laboratory of Veterinary Small Animal Internal Medicine, Cooperative Department of Veterinary Medicine, Faculty of Veterinary Medicine, Iwate University, Morioka 020-8550, Japan

**Keywords:** canine, cardiorenal syndrome, mitral regurgitation, pulmonary hypertension, velocity time integral, venous impedance index

## Abstract

**Background:**

While the predictive value of venous impedance index (VII) for evaluating renal congestion is reported in dogs with myxomatous mitral valve disease (MMVD), an ultrasonographic index of renal perfusion has not been reported.

**Hypothesis/Objectives:**

Low renal perfusion assessed by interlobar renal artery velocity time integral (VTI) is associated with a worse prognosis in dogs with MMVD. Combining VTI and VII provides additional prognostic information.

**Animals:**

Sixty-two client-owned dogs without heart disease evaluated for preanesthetic or general health assessment and 80 client-owned dogs with MMVD.

**Methods:**

Prospective cohort study. The VTI was measured by manually tracing the spectral velocity envelope of interlobar arteries. The primary endpoint was defined as the composite of cardiac-related death and renal-related death.

**Results:**

The VTI was corrected for the effect of heart rate (VTIc) because VTI and heart rate were negatively correlated in dogs without heart disease. In dogs with MMVD, the VTIc decreased with higher ACVIM stage. Dogs with lower VTIc (< 8.3) had significantly shorter survival times (median, not reached; 95% CI, 98 days-not reached; *P* < .001) than those with normal VTIc (median, not reached; 95% CI, not reached). In multivariable Cox analysis, VII remained significantly associated with outcome (HR, 1.24 per 0.1-unit increase; 95% CI, 1.07-1.42). Exploratory classification combining VII (<0.37 or ≥0.37) and VTIc (≥8.3 or < 8.3) identified 4 subsets with different survival outcomes.

**Conclusions and clinical importance:**

The VTIc might be associated with outcome in dogs with MMVD and combined assessment might provide additional exploratory prognostic information.

## Introduction

The kidneys and heart have close and complementary interactions. Therefore, dysfunction of either organ system can cause dysfunction of the other system.^1^ In human patients with heart failure, renal dysfunction is commonly observed and associated with higher morbidity and case fatality.^2^ Similarly, in dogs with myxomatous mitral valve disease (MMVD), the prevalence of renal disease increases with the severity of heart disease^3^^,^^4^ and concomitant renal disease is associated with significantly shorter survival times.^3^ In the pathophysiology of heart failure, renal congestion, and renal hypoperfusion are involved in renal damage.^5–7^ Renal congestion is caused by elevated right atrial pressure and lower right ventricular function, and renal hypoperfusion is related to a decrease in cardiac output in human patients with heart failure.^5–7^

Intrarenal Doppler ultrasonography (IRD) using pulsed-wave Doppler is useful for evaluating intrarenal hemodynamics, especially in the evaluation of renal congestion in human medicine.^8^ A discontinuous intrarenal venous flow (IRVF) pattern, which are interruptions of intrarenal venous blood flow within the cardiac cycle, is strongly associated with poorer clinical outcomes in human patients with heart failure.^8–10^ In veterinary medicine, a discontinuous IRVF pattern or an elevated venous impedance index (VII) obtained from renal venous flow have been observed in dogs with advanced MMVD.^11^ Furthermore, elevated VII was an independent predictor of worse prognosis in dogs with MMVD.^11^ Based on these findings, the assessment of renal congestion using IRD might be clinically useful for predicting a poor outcome in both human and dogs with heart disease. However, compared with renal congestion, limited information is currently available regarding assessment of renal hypoperfusion, particularly in dogs. Although the resistance index calculated from the renal artery waveform is used in assessing intrarenal hemodynamics, it was not associated with worse outcome unlike IRVF pattern in either human patients with heart failure or dogs with MMVD.^8^^,^^11^ The velocity time integral (VTI) of the renal interlobar artery in humans has the potential to assess intrarenal hypoperfusion.^9^ A lower in VTI is associated with lower blood pressure and reduced cardiac output, and is a poor prognostic factor for patients with heart failure.^9^ The combined assessment of VTI and IRVF patterns allowed for more detailed prognostic stratification.^9^

However, no study has yet focused on VTI of interlobar renal artery in dogs. Therefore, this study aimed to evaluate VTI and compare VTI in dogs with MMVD at different stages, and to investigate the prognostic value of VTI in dogs with MMVD. We hypothesize that VTI changes with the severity of disease and is associated with poor prognosis, and that the combined evaluation of VTI and VII would be a useful to estimate prognosis in detail in dogs with MMVD.

## Materials and methods

Client-owned dogs without heart disease (controls) and dogs with MMVD were prospectively enrolled from the Veterinary Teaching Hospital of Iwate University between November 2019 and November 2024. Dogs with clinically relevant systemic diseases, atrial fibrillation or ventricular tachycardia, or advanced chronic kidney disease (IRIS stage 3 or 4) were excluded. Dogs with MMVD were classified according to the American College of Veterinary Internal Medicine (ACVIM) consensus guidelines.^12^

Comprehensive echocardiography and IRD were performed in conscious dogs using the same ultrasonography system. Standard echocardiographic measurements, including the Mitral INsufficiency Echocardiographic (MINE) score, were obtained. Intrarenal arterial and venous Doppler signals were recorded from the left kidney. The VTI of the interlobar renal artery was measured by manual tracing of the Doppler spectral envelope, and the VII was calculated from intrarenal venous flow velocities Figure 1. Measurements were averaged over three cardiac cycles.

**Figure 1:**
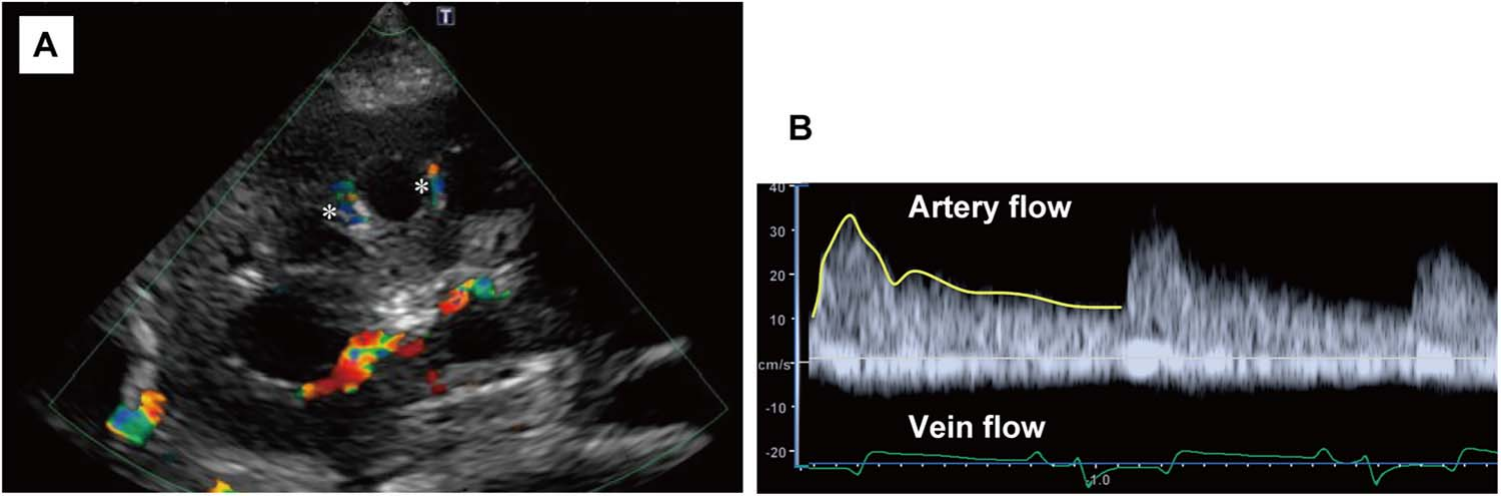
Images illustrating the technique used to perform intrarenal Doppler ultrasonography. (A) Color Doppler flow image from the left kidney. The sample volume was set in the interlobar vessels (*). (B) Pulsed-wave Doppler waveform from the interlobar vessels. The upward Doppler signal shows interlobar artery flow and the downward Doppler signal shows interlobar vein flow. Intrarenal venous flow shows a continuous pattern. The velocity time integral was measured as the area under the outermost portion of the spectral velocity envelope.

Outcome adjudication was performed by the same investigator who performed the ultrasonographic examinations by reviewing medical records and contacting referring veterinarians and dog owners; therefore, adjudication was not blinded to the IRD findings. The primary endpoint of this study was a composite of cardiac-related death and renal-related death (cardiorenal death). Dogs were censored if they were alive at the end of the study period, died owing to non-cardiorenal death, or underwent mitral valve repair.

Reference intervals for VTI were determined using a software add-on (Excel Reference Value Advisor add-on)^13^ in dogs without heart disease. Dogs with values below the lower limit of the reference interval were defined as the low VTI group, while the remaining dogs were defined as the normal VTI group. Dogs with values above the reported upper limit of the reference interval (VII > 0.37) were defined as the high VII group.^14^ Survival was evaluated using Kaplan–Meier analysis and Cox proportional hazards models. Both VTI and VII were analyzed as dichotomized and continuous variables. For further stratification, dogs were classified into 4 subsets based on VTI in combination with VII (subset 1-4). Because of the limited number of outcome events, only clinically relevant covariates (MINE score and plasma creatinine concentration) were included in multivariable models to reduce overfitting. Detailed methodology is provided in Appendix 1.

## Results

### Control groups

This study included 62 dogs from 16 breeds, with Toy poodle being the most frequently represented (*n* = 19), followed by Chihuahua (*n* = 12), Beagle (*n* = 8), mixed breeds (*n* = 4), Miniature Dachshund (*n* = 3), Pomeranian (*n* = 3). Median body weight was 4.0 (2.8-7.1) kg, and median age was 4.5 (1.0-7.3) years.

The mean VII value was 0.23 ± 0.05 (reference interval, 0.13-0.37). The VTI and heart rate were negatively correlated (ρ = −0.40, *P* = .001). Therefore, we corrected for the effect of heart rate as following previously reported formula^15^: corrected VTI (VTIc) = VTI/√ (R-R interval). There was no significant correlation between VTIc and heart rate (ρ = −0.16, *P* = 0.21). The median VTIc value was 13.2 (25th-75th percentiles, 10.8-15.2). Lower limit of reference interval was 8.3 and upper limit was 22.0. No significant correlation was found between VTIc and age, body weight, and mean blood pressure. The VTIc did not differ between males and females.

### M‌MVD groups

#### Characteristics

The selection process of the study sample is summarized in Figure 2. A total of 91 dogs were screened for eligibility. Of these, 11 were excluded due to predefined exclusion criteria, resulting in 80 dogs being included. Eighty dogs from 15 breeds, with Chihuahua being the most frequently represented (*n* = 18), followed by Toy poodle (*n* = 15), mixed breeds (*n* = 12), Cavalier King Charles Spaniel (*n* = 11), and Pomeranian (*n* = 6) were included. Median body weight was 4.7 (3.3-6.9) kg, and mean age was 11.4 ± 2.4 years. Mean systolic and diastolic blood pressure were 149 ± 19 and 92 ± 14 mmHg, respectively. Twenty-four dogs had systemic hypertension. Dogs were classified into stage B1 (*n* = 29), stage B2 (*n* = 31), stage C (*n* = 18), and stage D (*n* = 2). Dogs with stage C and stage D were combined into one group for statistical analysis, because there were only 2 dogs in stage D. Among the dogs classified as stage C and D, 4 had isolated RHF, 16 had a history of left-sided congestive heart failure, and 5 had a history of both left-sided and right-sided heart failure; thus, a total of 9 dogs showed ascites due to RHF. Seventeen dogs were classified as mild, 23 were moderate, 38 were severe, and 2 were late stage based on MINE score. Based on the probability of PH, 45 dogs were classified under the low probability, 12 dogs under the intermediate probability, and 23 dogs under the high probability. Forty-five dogs were prescribed pimobendan. The mean pimobendan dosage was 0.33 ± 0.10 mg/kg *q*12h. Angiotensin-converting enzyme inhibitors were administered in 31 dogs, including alacepril (*n* = 15), benazepril (*n* = 11), temocapril (*n* = 4), and enalapril (*n* = 1). Alacepril was administered at 2.53 ± 1.47 mg/kg/day and benazepril at 0.59 ± 0.24 mg/kg/day. Median temocapril dose was 0.17 mg/kg/day (range 0.11-0.20 mg/kg/day). Enalapril was administered in only one dog at a dose of 0.30 mg/kg/day. Twenty-four dogs were prescribed diuretics. Four dogs in the stage B2 were on diuretics prescribed by the referring hospital at the time of the initial examination. Furosemide and torasemide were administered in 18 and 6 dogs, respectively. Furosemide was administered at 4.42 ± 1.83 mg/kg/day, torasemide at 0.28 ± 0.12 mg/kg/day. Three dogs were prescribed sildenafil citrates. Sildenafil was administered at 2.0-3.0 mg/kg/day. Spironolactone was administered in 12 dogs (1.60 ± 0.46 mg/kg/day).

**Figure 2:**
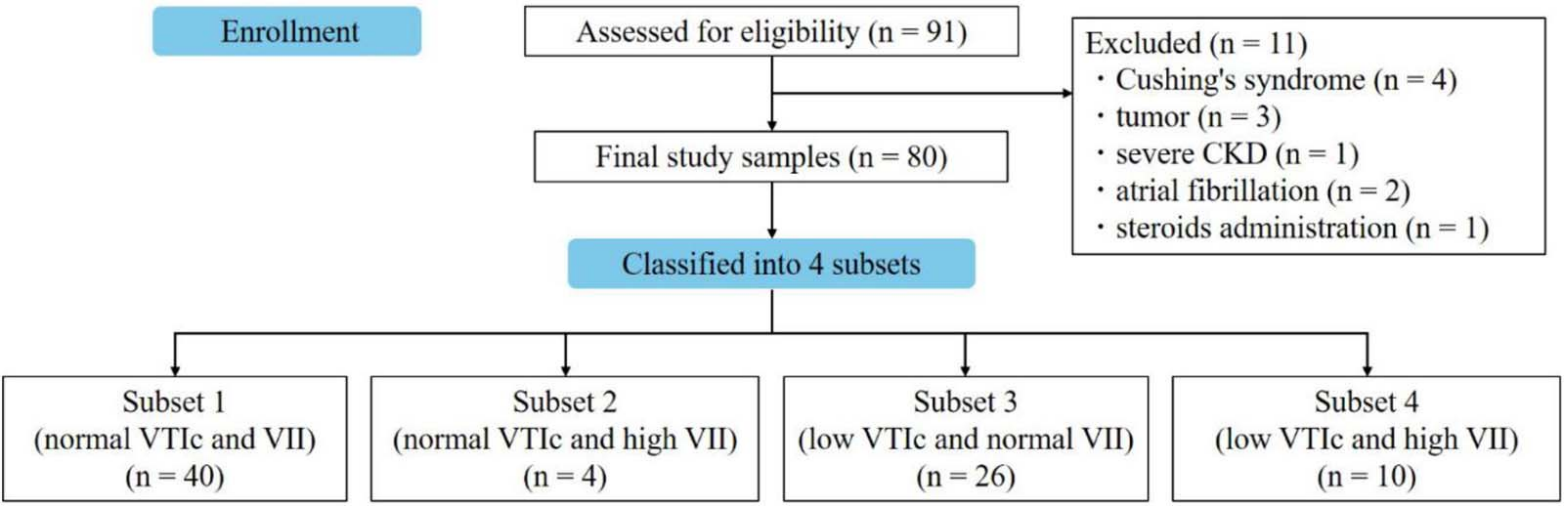
Flow diagram of case selection and inclusion in the study. A total of 91 dogs were initially assessed for eligibility. After exclusion of 11 dog, 80 dogs were included in the final analysis and classified into 4 subsets.

#### Intrarenal doppler ultrasonography findings

Dogs with VTIc < 8.3 were defined as the low VTIc group based on lower limit of reference interval. Thirty-six dogs were subdivided in low VTIc group, including 5 dogs classified as stage B1, 15 as stage B2, 15 as stage C, and 1 as stage D. In 14 dogs with VII > 0.37, 7 of those dogs had a VII > 0.37 without a discontinuous IRVF pattern. Dogs were subdivided in those showing a continuous (*n* = 73) and a discontinuous (*n* = 7) IRVF pattern. Five and 2 dogs showing a discontinuous IRVF pattern were stage C and D, respectively. All 7 dogs showing a discontinuous IRVF pattern showed ascites due to RHF. No significant differences in VTIc or VII were observed between dogs with isolated RHF and those with left-sided congestive heart failure. Representative image of IRD in dogs subdivided into 4 subsets based on VTIc and VII were shown in Figure 3. When categorizing into 4 subsets by combining the VTIc and VII evaluations, 40 dogs in the subset 1, 4 dogs in the subset 2, 26 dogs in the subset 3, and 10 dogs in the subset 4.

**Figure 3:**
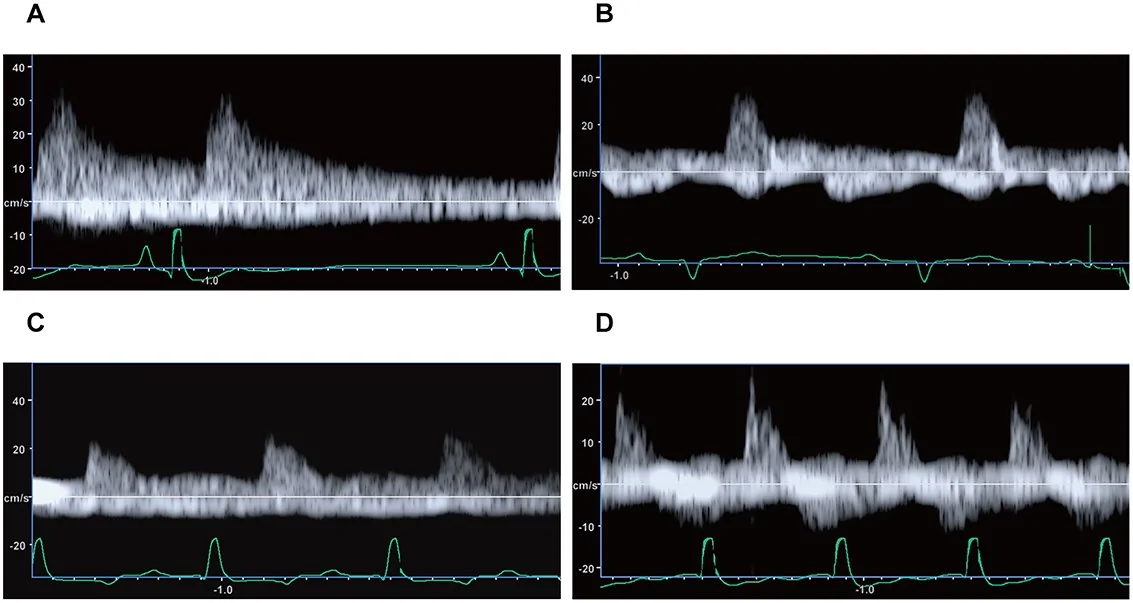
Representative image of intrarenal Doppler ultrasonography in dogs with myxomatous mitral valve disease subdivided into 4 subsets (subset 1-4). (A) The VII and VTIc was normal in the subset 1. (B) The value of VII was high, but VTIc was normal in the subset 2. (C) The value of VTIc was low, but VII was normal in the subset 3. (D) Both VII and VTIc was abnormal in the subset 4. Abbreviations: VII = venous impedance index; VTIc = corrected velocity time integral.

The VTIc was significantly lower in stage B2 (median, 8.35; 25th-75th percentiles, 7.20-10.12; *P* = .003) and stage C/D groups (median, 6.91; 25th-75th percentiles, 6.04-7.97; *P* < .001) than in stage B1 group (median, 11.75; 25th-75th percentiles, 9.49-14.31) and was also significantly lower in stage C/D groups than in stage B2 group (*P* = .003) (Figure 4A). The VII was significantly higher in stage C/D (median, 0.49; 25th-75th percentiles, 0.29-1.00) groups than in stage B1(median, 0.24; 25th-75th percentiles, 0.22-0.29; *P* < .001) and B2 group (median, 0.26; 25th-75th percentiles, 0.21-0.28; *P* < .001) (Figure 4B).

**Figure 4:**
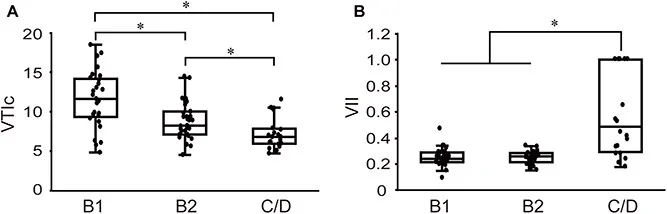
Box-and-whisker plots with overlaid individual data points of VTIc and VII in dogs with myxomatous mitral valve disease. (A) Corrected velocity time integral was significantly lower in stage B2 and stage C/D groups than in stage B1 group, and was also significantly lower in stage C/D groups than in stage B2 group. (B) Venous impedance index was significantly higher in ACVIM stage C dogs than in stage B1 and B2. ^*^P < .05.

The VTIc was significantly lower in high probability of PH group (median, 7.20; 25th-75th percentiles, 6.53-9.01) than in intermediate probability of PH group (median, 11.50; 25th-75th percentiles, 6.84-13.90; *P* = .03) and low probability of PH group (median, 9.80; 25th-75th percentiles, 7.51-12.30; *P* = .01). The VII was significantly higher in high probability of PH group (median, 0.39; 25th-75th percentiles, 0.28-1.00) than in intermediate probability of PH group (median, 0.25; 25th-75th percentiles, 0.21-0.28, *P* < .001) and low probability of PH group (median, 0.25; 25th-75th percentiles, 0.21-0.29, *P* < .001).

#### Correlation analysis

The VTIc showed fair negative correlation with plasma creatinine concentration (ρ = −0.33, *P* = .01). There was no significant correlation between VTIc and mean blood pressure or stroke volume index (SVI). The MINE score showed fair negative correlation with VTIc (ρ = −0.46, *P* < .001). The VTIc showed fair negative correlation with right ventricle (RV) Tei index (ρ = −0.25, *P* = .03), and fair positive correlation with right ventricular fractional area change (RV-FAC) (ρ = 0.37, *P* < .001). Significant fair negative correlations were found between VTIc and VII (ρ = −0.23, *P* = .04) (Figure 5).

**Figure 5:**
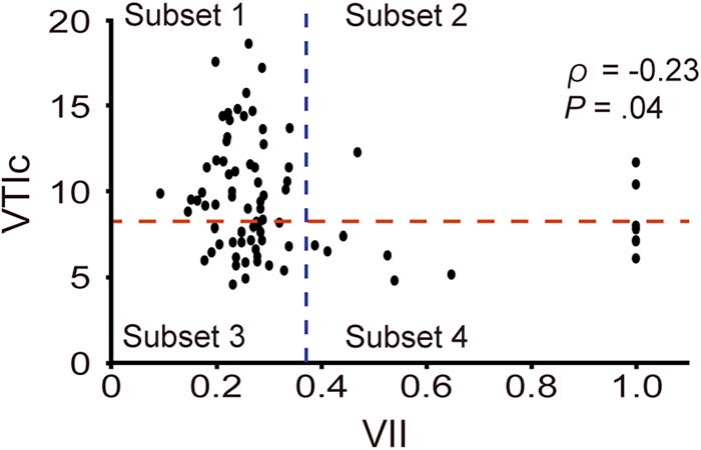
Correlation between VTIc and VII in dogs with myxomatous mitral valve disease. The VTIc was significantly correlated with VII (ρ = −0.23, *P* = .04). The red dashed line indicates the VTIc cutoff value (8.3), and the blue dashed line indicates the VII cutoff value (0.37). Abbreviations: VII = venous impedance index; VTIc = corrected velocity time integral.

#### Survival analysis

Median follow-up period was 476 days (95% CI, 7-1468 days). At the time when the survival information was collected (April 2025), 23 dogs (29%) met the primary endpoint (18 dogs [23%] died of cardiac-related causes and 5 dogs [6%] died of renal-related causes). None of the dogs was euthanized in the present study. Eleven dogs (14%) were considered to have died of other causes, and 44 dogs (55%) were still alive at the end of the study. Two dogs were censored on the day of mitral valve repair surgery (38 and 931 days from the time of diagnosis). The median survival time for all-cause death was 250 days (95% CI, 5-1118 days), while that for cardiorenal death was 193 days (95% CI, 5-460 days). The median follow-up time for dogs still alive was 704 days (95% CI, 170-1558 days).

The Kaplan–Meier analysis with log-rank test revealed the significant effects of VTIc and VII on survival (Table 1). Dogs with low VTIc had significantly shorter survival time than those with normal VTIc (*P* = .01) (Figure 6A). Dogs with VII > 0.37 had significantly shorter survival times than those with VII ≤ 0.37 (*P* < .001). Four dogs of subset 1 (4/40), 2 dogs of subset 2 (2/4), 9 dogs of subset 3 (9/26), and 8 dogs of subset 4 (8/10) met the primary endpoint. As shown in Figure 6B, MMVD dogs with low VTIc and high VII (subset 4) had significantly shorter survival time than those with normal VTIc and VII (subset 1) (*P* < .001) and low VITc and normal VII (subset 3) (*P* = .002).

**Table 1:** Kaplan–Meier analysis with log-rank test in 80 dogs with myxomatous mitral valve disease.

**Subgroup**	***n***	**Median survival time (day)**	**95% CI**	
**VII**				
**>0.37**	14	180	55-ND	<.001
**≤0.37**	66	ND (>1558)	ND	
**VTIc**				
**<8.3**	36	449	166-ND	<.001
**≥8.3**	44	ND (>1558)	ND	
**Combination of VII and VTIc**				
**Subset 1 (normal VII and VTIc)**	40	ND (>1558)	ND	<.001
**Subset 2 (high VII and normal VTIc)**	4	ND	277-ND	
**Subset 3 (normal VII and low VTIc)**	26	ND (>1558)	333–ND	
**Subset 4 (high VII and low VTIc)**	10	108	7-193	

If half of the dogs or more did not reach the primary endpoint during the follow-up period, median survival time was defined as not determinable (ND).

Abbreviations: ND = not determinable; VII = venous impedance index; VTIc = corrected velocity time integral.

**Figure 6:**
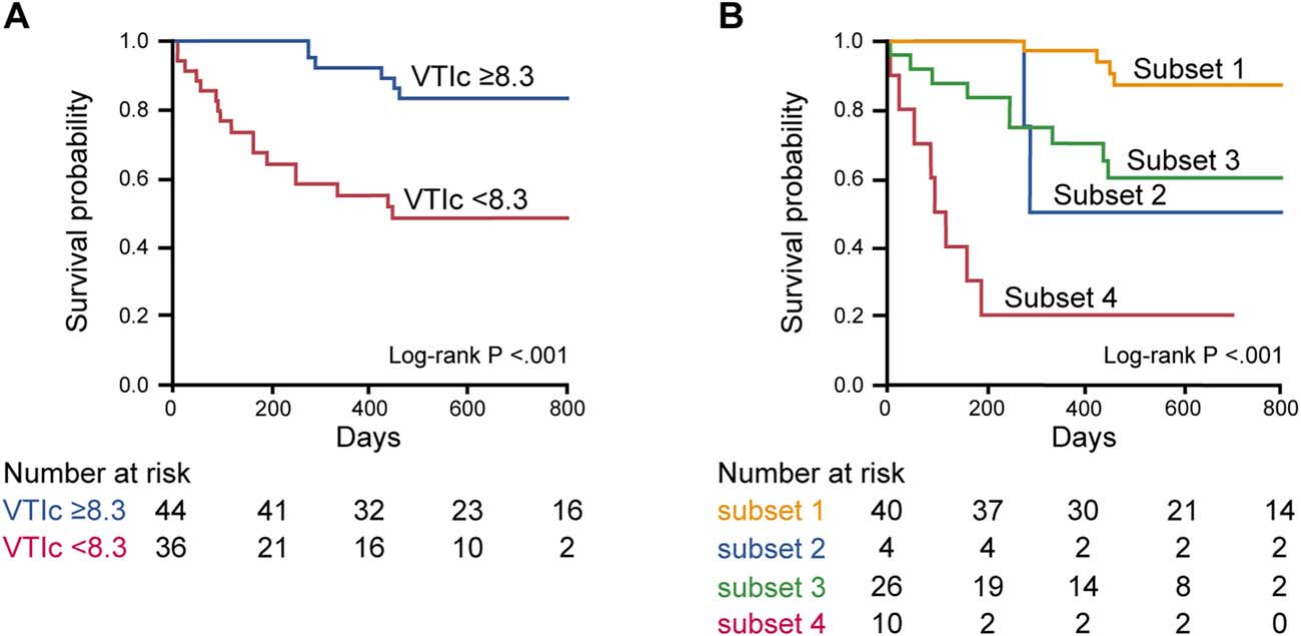
Kaplan–Meier analysis of survival in dogs with myxomatous mitral valve disease. (A) Dogs with low VTIc (≤8.3) had worse outcomes than those with normal VTIc. (B) Dogs in the subset 4 had significantly shorter survival time than those in the subset 1 and subset 3. Numbers at risk are shown below each Kaplan–Meier plot. Abbreviations: VII = venous impedance index; VTIc = corrected velocity time integral.

In the univariable Cox proportional hazard analysis, VTIc < 8.3, VII > 0.37, and the combination of VTIc and VII were associated with cardiorenal death in dogs with MMVD (Table 2). Even after adjustments for MINE score and plasma creatinine concentrations, the combination of VTIc and VII still remain associated with the primary endpoint remained significant (*P* < .001) (Table 2). When analyzed as continuous variables, VTIc was significant in the univariable Cox analysis but not after multivariable adjustment, whereas VII remained significant in both analyses. Given the limited number of events, additional multivariable adjustment with multiple clinical covariates was not performed because of concerns regarding model instability and overfitting.

**Table 2:** Univariable and multivariable cox proportional hazard analysis for the association with the cardiorenal death in 80 dogs with myxomatous mitral valve disease.

**Variable**	**Unadjusted**		**Adjusted**	
	**HR (95% CI)**	***P***	**HR (95% CI)**	***P***
**MINE score (per 1 score)**	1.62 (1.33-2.01)	<.001		
**plasma creatinine level (per 0.1 mg/dl)**	1.14 (1.01-1.27)	.03		
**IRD variables**				
**VTIc (per 1 unit)**	0.76 (0.62-0.89)	<.001	0.87 (0.69-1.06)	.19
**VTIc < 8.3**	2.97 (1.30-6.74)	.01	1.84 (0.68-4.98)	.23
**VII (per 0.1 unit)**	1.37 (1.20–1.54)	<.001	1.24 (1.07–1.42)	.003
**VII > 0.37**	5.78 (2.51-13.30)	<.001	2.67 (1.04-6.88)	.04
**Combination of VTIc and VII**				
**Subset 2 (normal VTIc and high VII)^a^**	5.81 (1.69-20.00)	.01	3.72 (0.81-17.05)	.09
**Subset 3 (low VTIc and normal VII)^a^**	2.66 (0.89-7.91)	.08	3.85 (1.11-13.39)	.03
**Subset 4 (low VTIc and high VII)^a^**	11.18 (3.70-33.76)	<.001	11.20 (3.02-41.48)	<.001
**Subset 4 (low VTIc and high VII)^b^**	1.92 (0.54-6.88)	.31	3.01 (0.99-9.11)	.05
**Subset 4 (low VTIc and high VII)^c^**	4.21 (1.35-13.19)	.01	2.91 (1.57-5.37)	<.001

Multivariable Cox proportional hazard analyses included 3 variables; MINE score, plasma creatinine level, and each of IRD variables.

Abbreviations: HR = hazard ratio; IRD = intrarenal Doppler ultrasonography; MINE = Mitral INsufficiency Echocardiographic; VII = venous impedance index; VTIc = corrected velocity time integral

^a^vs. subset 1 (normal VTIc and VII) ^b^vs. subset 2 ^c^vs. subset 3.

## Discussion

In the present study, VTIc was lower with higher MINE score and progression of ACVIM stage, and decreased even in preclinical stage. Decreased VTIc was associated with deterioration of echocardiographic indices of both the left heart and the right heart. Dogs with lower VTIc had a significantly shorter survival time than those with normal VTIc, although the association between continuous VTIc and outcome was no longer significant after multivariable adjustment. The exploratory 4-subset classification based on dichotomized VTIc and VII suggested that combined assessment of renal perfusion and congestion might provide additional prognostic information.

A significant negative correlation was observed between heart rate and VTI in control groups. The VTI corresponds to the area of the renal artery waveform that is observed continuously over one cardiac cycle, and is therefore thought to be high in cases with a longer R-R interval (ie, a low heart rate). As respiratory arrhythmia is common in dogs, VTI measurements might vary depending on the heart rate used for measurement even within the same patient. Therefore, we considered that a correction based on the R-R interval was necessary. In this study, no significant correlation was found between HR-corrected VTI (VTIc) and age, body weight, and mean blood pressure. This study evaluates the relationship between VTI and characteristics in dogs.

A negative correlation was observed between VTIc and the MINE score in this study. Furthermore, VTIc gradually decreased as the stage of ACVIM progressed and was low even in stage B2. These findings indicate that reduced renal perfusion progresses as MMVD becomes more severe. In human patients with heart failure, reduced VTI was associated with hypotension and reduced cardiac output.^9^ However, there was no correlation between VTIc and blood pressure or stroke volume in this study. This difference might be due to the severity, chronicity, or type of heart disease. While study of human patients included decompensated heart failure patients including various type of heart disease, the majority of dogs were preclinical stage MMVD (ACVIM stages B1 and B2) in this study (58/71 dogs).

A fair negative correlation was observed between VTIc and plasma creatinine concentration, suggesting only a limited association between renal perfusion indices and renal function markers in dogs with MMVD. Persistent renal hypoperfusion might contribute to renal cellular injury and renal dysfunction through mechanisms involving adenosine triphosphate depletion, epithelial injury and death, and activation of inflammatory processes.^16^ Therefore, intrarenal hypoperfusion is proposed as one potential mechanism contributing to renal dysfunction in human patients with heart failure.^5^^,^^7^ In this study, low VTIc showed only a fair association with renal function indices; however, these findings might partially support the possibility that intrarenal hypoperfusion contributes to renal dysfunction in some dogs with MMVD similar to that seen in human patients with heart failure. Nevertheless, the clinical importance of this relationship remains uncertain and requires further investigation. Since elevated plasma blood urea nitrogen (BUN) and creatinine values can also be caused by renal abnormalities separate from cardiac disease, dogs with severe CKD (ie, IRIS stage 3 or 4) were excluded in this study, and dogs whose renal function decline was thought to be mainly due to MMVD were included to investigate the interaction between the kidney and the heart. However, glomerular filtration rate was not measured in this study, and the possibility that mild CKD altered VTIc cannot be excluded.

In dogs with MMVD, VII significantly elevated only in stage C. In human patients with heart failure, renal congestion associated with elevated right atrial pressure and impaired right ventricular function is considered an important component of cardiorenal syndrome. Similarly, we previously demonstrated that right atrial enlargement and right ventricular dysfunction are associated with increased VII in dogs with advanced MMVD.^11^ These abnormalities are commonly observed in dogs with RHF. Therefore, the higher in VII observed in stage C dogs might reflect the development of venous congestion associated with progression to RHF. Dogs exhibiting a discontinuous IRVF pattern (VII = 1.0) showed clinical signs consistent with RHF. However, regarding IRD profile in dogs with ascites, it is possible that increased intra-abdominal pressure caused by ascites influence the IRD profile.^17^ Regarding VTIc, fair but significant correlations were observed with indices of RV function, suggesting that impaired RV function may also contribute to reduced renal perfusion. In contrast to VII, intrarenal perfusion assessed by VTIc was reduced even in the preclinical stage. This finding suggested that intrarenal hypoperfusion (ie, low values of VTIc) and renal congestion (ie, high values of VII), behave differently and reflect different pathological conditions. Therefore, assessment of both VTIc and VII may be potentially useful for management of dogs with MMVD.

In the present study, dogs with a low VTIc had significantly shorter survival time than those with a normal VTIc. When VTIc was analyzed as continuous variables, VTIc was significantly associated with outcome only in the univariable analysis, and this association was not apparent after adjustment. These findings suggest that renal venous congestion assessed by VII might have a more robust prognostic association than renal perfusion assessed by VTIc, whereas the relationship between VTIc and outcome may be influenced by disease severity, renal function, or other clinical factors. In contrast, VII significantly associated with cardiorenal death in both univariable and multivariable Cox analyses. In a previous study of patients with heart failure, the combining IRVF pattern and VTI into 4 subsets allowed stratification of clinical outcomes.^9^ In this study, we also attempted to compare the prognosis of dogs with MMVD among 4 subsets defined by combining VTIc and VII. Abnormalities in both VTIc and VII were associated with cardiorenal death (hazard ratio, 11.2 vs. subset 1; 2.9 vs. subset 3) in dogs with MMVD. This finding suggests that that in dogs with MMVD, both renal congestion and hypoperfusion might be associated with prognosis, and that evaluating both VII and VTIc enables more detailed prognostic stratification. As mentioned above, intrarenal hypoperfusion and renal congestion may behave differently and reflect different pathological conditions in dogs with MMVD, resulting in a detailed stratification of prognosis. The study results partially consistent with our first hypothesis in that VTI is associated with poor prognosis and that the combined evaluation of VTIc and VII might be potentially useful for more detailed prognostic assessment in dogs with MMVD. The 4-subset classification based on VTIc and VII may provide a clinically intuitive framework for describing the balance between renal perfusion and venous congestion and reflect a classification previously proposed in human patients with heart failure. Nevertheless, because VTIc was not independently associated with outcome after adjustment when analyzed as a continuous variable, the findings from the 4-subset analysis should be regarded as exploratory. In addition, because medical treatment was not standardized and may have influenced both renal hemodynamics and clinical outcome, these associations should be interpreted cautiously. From a therapeutic perspective, this approach might help characterize different hemodynamic phenotypes. The combined assessment of VTIc and VII might help characterize different intrarenal hemodynamic phenotypes. In human patients with heart failure, reductions in VII after diuretic therapy are reported, suggesting that intrarenal venous Doppler indices might reflect changes in congestion status during treatment.^18^^,^^19^ However, the present study did not evaluate treatment response or treatment selection, and whether these Doppler indices can be used to guide treatment in dogs with MMVD remains hypothetical and requires prospective investigation.

The present study had several limitations that need to be addressed. Since the same investigator performed the Doppler measurements and adjudicated outcomes, outcome assessment was not blinded to the IRD findings, and ascertainment bias cannot be excluded. The reference interval for VTIc was established from only 62 dogs without heart disease. Although this cohort was sufficient for exploratory analysis, it did not meet the recommendations for establishing reference intervals. Therefore, the proposed reference interval and cut-off values should be interpreted with caution and require validation in a larger population. The number of dogs reaching primary endpoint was small. Furthermore, the number of dogs subdivided into some subsets (2 and 4) was small, which may have reduced statistical power and increased the risk of model overfitting in the multivariable Cox analyses. Although the number of covariates was restricted to clinically relevant variables, the hazard ratio estimates (particularly for subset 2) showed wide confidence intervals, indicating limited precision and substantial uncertainty in the estimated effect sizes. Because of the limited number of primary endpoint events and the small sample sizes in some subsets, the prognostic findings should be considered exploratory and require confirmation in larger prospective studies. Another limitation of this study is that the heart rate correction formula used for VTIc was adapted from a previous pediatric human study and has not been formally validated in dogs with MMVD. Although the correction eliminated the significant correlation between VTI and heart rate in the present population, the optimal method for heart rate correction in conscious dogs, especially those with sinus respiratory arrhythmia, remains unclear and warrants further investigation. The association of ascites on data acquisition during renal Doppler examinations was not specifically evaluated in this study. In our opinion, the effect is likely limited; however, it remains unclear whether the presence of ascites affected the technical quality of the measurements or the data acquisition. While plasma creatinine concentration and BUN were used as renal function test in the present study, measurement of glomerular filtration rate (ie, inulin clearance) is needed to better define the relationship between renal function and VTI. In this study, disease severity and medical treatment was heterogeneous among the enrolled dogs. Dogs across ACVIM stages B1 through D were included in the analyses, and treatment protocols were not standardized during the follow-up period. In particular, medications such as pimobendan, diuretics, and pulmonary vasodilators may have influenced cardiac output and renal perfusion, and ultimately clinical outcome. Because medication use and ACVIM stage were not fully incorporated into the multivariable models, residual confounding may have affected the observed associations between IRD variables and prognosis. Therefore, the results should be interpreted cautiously. Another limitation of this study is that systemic hypertension was not an exclusion criterion. Although no significant correlation was observed between mean blood pressure and the intrarenal Doppler indices, and dogs with advanced chronic kidney disease (IRIS stages 3 and 4) were excluded, systemic hypertension might still have influenced renal hemodynamics and clinical outcomes. Therefore, its potential impact on the observed associations cannot be completely excluded.

## Conclusions

VTIc declined with the progression of MMVD even in the preclinical stage. Intrarenal hypoperfusion, as assessed by VTIc, was associated with adverse clinical outcome in dogs with MMVD.

## Off-label antimicrobial declaration

Authors declare no off-label use of antimicrobials.

## Institutional animal care and use committee or other approval declaration

Approved by the Animal Research Committee of Iwate University (Approval No. A201941).

## Appendix: Detailed materials and methods

### Control groups

Dogs without heart disease underwent complete physical examination and echocardiography to confirm normal cardiac anatomy and function. Dogs with clinically relevant systemic diseases, including respiratory disease, neoplasia, adrenal or thyroid disorders, or medications affecting cardiovascular or renal function (eg, corticosteroids), were excluded. Dogs of any age and breed were included in the study. All dogs underwent echocardiography as either preanesthesia examinations or general health assessment. Dogs with International Renal Interest Society stage 2, 3, and 4 chronic kidney disease diagnosed as a serum creatinine concentration > 1.4 mg/dL^20^ were excluded. Complete blood count, serum biochemistry, thoracic radiography, and indirect blood pressure measurement (petMAP Graphic II; Cardio Command Inc., Tampa, FL, USA) were performed when necessary to confirm eligibility. Mean blood pressure was obtained as the average of 5 consecutive measurements as per recommended guidelines.^21^

### Myxomatous mitral valve disease dogs

Dogs were diagnosed with MMVD based on characteristic echocardiographic findings, including mitral valve leaflet thickening and/or mitral valve prolapse in B-mode accompanied by mitral regurgitation in color Doppler imaging. Dogs of any age were included. Dogs with clinically relevant systemic diseases including any tumors, adrenal and thyroid dysfunction, International Renal Interest Society stage 3 and 4 chronic kidney disease (a plasma creatinine concentration > 2.8 mg/dL),^20^ atrial fibrillation, ventricular tachycardia, or medications affecting cardiovascular or renal function were excluded. Systemic hypertension was not an exclusion criterion. Dogs with MMVD were classified into stage B1, B2, C, or D based on ACVIM consensus statement guidelines^12^ based on thoracic radiography and echocardiography. Stage C included MMVD dogs with current or past clinical and radiographic evidence of cardiogenic pulmonary edema or right-sided congestive heart failure (RHF). The probability of PH was examined using echocardiography based on ACVIM consensus statement guidelines^22^ and were classified into high probability, intermediate probability and low probability of PH. Plasma BUN and creatinine concentrations were measured in plasma after centrifugation using an automated biochemical analyzer (Catalyst One; IDEXX Laboratories Inc, Westbrook, ME). Feed was withheld from all dogs for 12 h before blood sampling.

### Echocardiographic measurements

The echocardiographic examination was performed one experienced echocardiographer (TM) using a commercially available ultrasound unit (Aplio a Verifia; Canon Medical Systems Corporation, Tochigi, Japan) with a sector transducer frequency ranging between 2.8 and 6.2 MHz (PST-50BT; Canon Medical Systems Corporation, Tochigi, Japan). All dogs were positioned in the left and right lateral recumbency without sedation. A simultaneous electrocardiogram trace (lead II) was recorded.

The stroke volume was calculated by multiplying the VTI of the pulmonary artery Doppler flow by the pulmonary artery luminal area. The pulmonary artery Doppler flow was recorded from a left parasternal short-axis view with sample volume placed just distal to the pulmonary valve and manually traced to obtain the time velocity integral. The stroke volume was normalized according to the following equation: SVI = stroke volume/body surface area.^23^^,^^24^ Body surface area was calculated as follows: body surface area = 10.1 × (body weight)^2/3^/10.^4^ Left ventricular dimensions were measured from a right parasternal short-axis view at the level of the chordae tendineae using the M-mode. The normalized dimensions was calculated as per the following equation^25^: left ventricular end-diastolic diameter normalized for body weight = LV end-diastolic internal diameter/(body weight)^0.294^. Fractional shortening was calculated as per the following equation: fractional shortening = (LV end-diastolic diameter—LV end-systolic diameter)/LV end-diastolic diameter × 100%. The left atrium and aorta diameters were obtained from a right parasternal short-axis view at the level of the left atrium, and the left atrium to aorta root diameter ratio was calculated.^26^ The transmitral early diastolic wave velocity was recorded in pulsed-wave Doppler with a left apical 4-chamber view. MINE score was calculated based on the values of left ventricular end-diastolic diameter normalized for body weight, fractional shortening, left atrium to aorta root diameter ratio, and transmitral early diastolic wave velocity.^27^ The RV Tei index was calculated as the sum of the isovolumic time divided by the ejection time using tissue Doppler.^28^ The RV end-diastolic area and end-systolic area were obtained by tracing the RV endocardium in end-diastole and end-systole from the annulus to apex with the RV-focused apical 4-chamber view optimized for the right heart, which included the RV apex, and the RV-FAC was calculated as (RV end-diastolic area—RV end-systolic area)/RV end-diastolic area × 100%. The right atrial (RA) area was measured by planimetrically tracing end-systole from the lateral aspect of the tricuspid annulus to the septal aspect, excluding the inferior and superior vena cava and RA appendage, from a RV-focused left apical 4-chamber view. The RA area was normalized according to the following equation: normalized RA area (nRAA) = RA area/body surface area.^29^

### Intrarenal Doppler ultrasonographic measurements

Intrarenal blood flow was recorded using pulsed-wave Doppler in right lateral recumbency without sedation using the same ultrasound system and transducer as echocardiography. Interlobar arteries and veins were detected using color Doppler imaging (velocity range, 16.0-19.2 cm/s). The sample volume (1.5-2.5 mm) was set at the interlobar vessels. The interlobar arteries and veins waveforms were recorded simultaneously (Figure 1). The VII were calculated by measuring maximum and minimum flow velocity as follows^8^: VII = (maximum flow velocity − minimum flow velocity)/maximum flow velocity from interlobar veins. The average of 3 measurements during sinus rhythm was used. Intrarenal venous waveforms were divided into continuous and discontinuous IRVF patterns. The discontinuous IRVF pattern was defined as having a minimum velocity of 0.^12^ The VTI was measured by manually tracing the spectral velocity envelope of interlobar arteries.^9^ All measurements were averaged over 3 cardiac cycles during the sinus rhythm. The R-R interval of the cardiac cycle measured VTI was also measured.

### Outcome assessment

Survival time was calculated as the interval from the day at the inclusion to either the day of death or closing time of the study (November 2024). Composite endpoint was selected because the objective of the study was to evaluate the prognostic significance of renal hemodynamic abnormalities in dogs with MMVD within the context of cardiorenal interaction. In dogs with advanced MMVD, cardiac dysfunction, venous congestion, renal hypoperfusion, and renal dysfunction are closely interconnected pathophysiological processes, and differentiation between cardiac- and renal-related causes of death is often clinically challenging. Cardiac-related death was included spontaneous death secondary to cardiogenic pulmonary edema or progressive right heart failure, euthanasia because of refractory left- and right-sided congestive heart failure (dyspnea, ascites, or pleural effusion) or sudden death when no other cause was evident. Renal-related death was defined as spontaneous death secondary to progressive azotemia when no abnormality of respiratory status and RHF was evident.

### Statistical analysis

Statistical analysis was performed using JMP version 14.0 (SAS Institute Inc., Cary, NC). Normal distribution of the data was confirmed by the Shapiro–Wilk test. Continuous data were reported as the mean ± SD for normally distributed data and the median (interquartile range) for nonnormally distributed data. Multiple group comparisons were performed with Tukey’s HSD test for normally distributed data and with Steel Dwass test for nonnormally distributed data. A Spearman correlation analysis was performed to determine the relationships between VTI and the clinical and echocardiographic variables. Correlation was reported as indicating poor to very strong correlation: < 0.2 is considered as poor, 0.21-0.60 as fair, 0.61-0.80 moderate, and > 0.8 as very strong.^30^

Survival analyses were performed using Kaplan–Meier analysis and compared using the log-rank test to evaluate the relationship between IRD indices and cardiorenal death. If half of the dogs or more did not reach the primary endpoint during the follow- up period, median survival time was defined as not determinable (ND). An exploratory 4-subset classification was subsequently constructed by combining VTI and VII categories (subset 1-4): subset 1, normal VTI and normal VII; subset 2, normal VTI and high VII; subset 3, low VTI and normal VII; subset 4, low VTI and high VII. For the multivariable Cox proportional hazards models, the MINE score was selected as a comprehensive indicator of MMVD severity based on previous prognostic studies,^27^ whereas plasma creatinine concentration was included because renal dysfunction has been associated with outcome in dogs with MMVD.^3^ Variables closely related to disease severity, including ACVIM stage and pulmonary hypertension probability, were not simultaneously included to avoid model instability and collinearity. The Cox proportional hazard model assumption was checked via the Schoenfeld residual.

The threshold for significance was set at *P*-value < .05.

## Abbreviations

ACVIM: American College of Veterinary Internal Medicine
CI: confidence interval
IRD: intrarenal Doppler ultrasonography
IQR: interquartile range
IRVF: intrarenal venous flow
MINE: Mitral INsufficiency Echocardiographic
MMVD: myxomatous mitral valvular disease
ND: not determinable
PH: pulmonary hypertension
RV: right ventricle
RV-FAC: right ventricular fractional area change
VII: venous impedance index
VTI: velocity time integral
VTIc: corrected velocity time integral
